# Association between Ischemic Stroke and Iron-Deficiency Anemia: A Population-Based Study

**DOI:** 10.1371/journal.pone.0082952

**Published:** 2013-12-09

**Authors:** Yen-Liang Chang, Shih-Han Hung, Wells Ling, Herng-Ching Lin, Hsien-Chang Li, Shiu-Dong Chung

**Affiliations:** 1 Department of Otolaryngology Head and Neck Surgery, Cathay General Hospital, Taipei, Taiwan; 2 School of Medicine, Fu-Jen Catholic University, Taipei, Taiwan; 3 Department of Otolaryngology, Taipei Medical University Hospital, Taipei, Taiwan; 4 Department of Psychology, Saint Louis University, Saint Louis, Missouri, United States of America; 5 Sleep Research Center, Taipei Medical University Hospital, Taipei, Taiwan; 6 School of Health Care Administration, Taipei Medical University, Taipei, Taiwan; 7 Division of Urology, Department of Surgery, Far Eastern Memorial Hospital, New Taipei City, Taiwan; University Medical Center Utrecht, Netherlands

## Abstract

**Background:**

Very little is known about the relationship between non-sickle cell anemia and stroke. The purpose of this study is to evaluate the association of iron-deficiency anemia (IDA) with stroke based on a nationwide coverage database in Taiwan.

**Methods:**

The case-control study subjects were obtained from the Taiwanese Longitudinal Health Insurance Database 2000. We included 51,093 subjects with stroke as cases and randomly selected 153,279 controls (3 controls per case) in this study.Separate conditional logistic regression analyses were used to calculate the odds ratio (OR) for having been previously diagnosed with IDA between cases and controls.We further analyzed the association between stroke and IDA by stroke subtype.

**Results:**

Results showed that 3,685 study subjects (1.81%) had been diagnosed with IDA prior to the index date; of those subjects, 1,268 (2.48%) were cases and 2,417 (1.58%) were controls (*p*<0.001). Conditional logistic regression shows that the OR of having previously received an IDA diagnosis among cases was 1.49 (95% CI: 1.39~1.60; *p* < 0.01) that of controls after adjusting for monthly income, geographic region, hypertension, diabetes, coronary heart disease, atrial fibrillation, heart failure, hyperlipidemia, tobacco use disorder, and alcohol abuse/alcohol dependency syndrome. Furthermore, the adjusted OR of prior IDA for cases with ischemic stroke was found to be 1.45 (95% CI: 1.34~1.58) compared to controls. However, we did not find any significant relationship between IDA and subarachnoid/intracerebral hemorrhage even adjusting for other confounding factors (OR=1.17, 95% CI=0.97~1.40).

**Conclusion:**

There is a significant association between prior IDA and ischemic stroke.

## Introduction

Cerebrovascular accident (CVA), or stroke, is the rapid loss of brain function due to a disturbance in the blood supply to the brain. According to the latest report from the Centers for Disease Control and Prevention, mortality from stroke was the fourth leading cause of death in the United States in 2008, and stroke was a leading cause of long-term severe disability [1]. Nearly half of older stroke survivors were noted to experience moderate to severe disability [2]. Care for stroke survivors has been estimated to cost $18.8 billion in health care expenses within the United States during 2008, in addition to $15.5 billion as a result of lost productivity and premature mortality [3]. The disease has been linked to several possible causes, including ischemia (lack of blood flow) caused by blockage (thrombosis, arterial embolism), or a hemorrhage [4]. Approximately 80 percent of strokes are due to ischemic cerebral infarction and 20 percent to brain hemorrhage.

Anemia is the most common disorder of the blood and had been proved to be highly related to the cardiovascular diseases as well as cerebrovascular accidents [5-9]. However, only the subtype of sickle cell anemia was reported to be highly associated with CVA [10-13].With increasing evidence indicating that underlying genetic and molecular mechanisms are responsible for these accidents, the pathogenesis of stroke or other morbidities in sickle cell anemia appears to be quite complex and different from the more commonly seen iron deficiency anemia (IDA). 

Very little were known about the relationship between non-sickle cell anemia and stroke. Perhaps the most related studies connecting these two problems are those focusing on the correlation between anemia and negative outcomes after ischemic stroke [14-16].The impact of hematocrit on long-term cardiovascular risk profile and overall survival was investigated in the Framingham study and surprisingly the authors found that most subjects with low hematocrit died of causes other than cardiovascular disease, whereas cardiovascular deaths contributed a larger proportion to the mortality of subjects in the high hematocrit group [17]. These findings appear to contradict the field’s general view on the relationship between the IDA and the stroke. Given this, the study aims to better understand the association of IDA and stroke based on a sample from the nationwide coverage database in Taiwan.

## Methods

### Database

Data for this study were retrieved from the “Longitudinal Health Insurance Database (LHID2000)”. The LHID2000 includes all medical claims and registration files for 1,000,000 enrollees under the Taiwan National Health Insurance (NHI) program, which was initiated in 1995. These 1,000,000 enrollees of the LHID2000 were randomly selected from all enrollees listed in the 2000 Registry of Beneficiaries (*n*=23.72 million) under the NHI program. The Taiwan National Health Research Institute as well as some researchers have demonstrated the high validity of the LHID2000 [18,19].Furthermore, hundreds of papers employing the LHID2000 have been published in internationally peer-reviewed journals [20].

### Study Sample

Study subjects were obtained from the LHID2000. Study cases were choose from subjects who had received their first-time diagnosis of stroke (ICD-9-CM codes 430-438) in an ambulatory care visit (including outpatient departments of hospitals and clinics) or hospitalization from January 1, 2003 to December 31, 2011. We identified 51,238 subjects who fit the criteria. Subjects who were under the age of 18 years (*n*=145) were excluded in order to limit the study to an adult population. We then assigned the date of their first stroke diagnosis as the index date. Ultimately, 51,093 subjects with stroke were included as cases in this study.

For the control group, we first identified all subject ≥18 years from the LHID2000, and then excluded enrollees who had a history of stroke since the initiation of the NHI in 1995. From the remaining subjects, 153,279 controls (3 controls per case) were randomly selected and then matched with the study subjects by sex, age group (18~39, 40~44, 45~49, 50~54, 55~59, 60~64, 65~69, and >69 years), and index year by SAS proc surveyselect program. We then assigned the date of their first visit of a physician occurring during that matched year as the index date for controls. For cases, the year of the index date was the year in which the cases received their first diagnosis of stroke, for controls, the year of the index date was simply a matched year in which controls visited a physician. As a result, 204,372 study subjects including 51,093 cases and 153,279 controls were in this study.

### Exposure assessment

This study identified IDA cases based on ICD-9-CM codes 280, 280.0, 280.1, 280.8, and 280.9. In order to increase the validity of IDA diagnoses, this study only included cases that have had at least two diagnoses of IDA in their medical claims prior to their index date as IDA cases. According to our clinical guidelines and health insurance regulations under the Taiwan NHHI program, patients suspected to have IDA might receive a diagnosis of unspecified anemia (ICD-9-CM code 285) in their first visit. However to have a definite diagnosis of IDA (ICD-9-CM code 280) the patient must be confirmed by receiving the laboratory test (decreased serum iron and ferritin, increased TIBC). Therefore, we believe that the diagnosis of IDA remains fairly reliable.

### Statistical Analysis

We used the SAS statistical package (SAS System for Windows, vers. 8.2, Cary NC, USA) to perform all statistical analyses. Chi-squared tests were conducted to examine the differences between cases and controls in terms of sociodemographic characteristics and medical co-morbidities. Medical co-morbidities included hypertension, diabetes, coronary heart disease (CHD), heart failure, atrial fibrillation, hyperlipidemia, tobacco use disorder, obesity, and alcohol abuse/alcohol dependency syndrome. These co-morbidities have all been reported to be risk factors for stroke. In addition, similar multivariate-model has been used to estimate the risk of stroke by prior studies [21-23]. Furthermore, separate conditional logistic regression analyses (conditioned on sex, age group, and index year) were used to calculate the odds ratio (OR) for having been previously diagnosed with IDA between cases and controls. We further analyzed the association between stroke and IDA by stroke subtype. The stroke subtypes are classified into subarachnoid/intracerebral hemorrhage (ICD-9-CM code 430 and 431), ischemic stroke (ICD-9-CM codes 433, 434 and 435) and unspecified strokes (ICD-9-CM codes 436 and 437). Odds ratios (ORs) along with 95% CIs were used to present the odds of prior IDA, with a two-sided *p* value of <0.05 considered statistically significant.

## Results

Of the 51,093 cases and 153,279 controls, the mean age was 64.1 ± 15.0 years; 64.1 ± 14.9 and 64.0± 15.0 for cases and controls, respectively. After matching for sex and age group, table 1 shows that cases were more likely to have income <NT$15,841 and resided in eastern part of Taiwan than controls. In addition, cases had a higher prevalence of medical co-morbidities including hypertension, diabetes, CHD, atrial fibrillation, heart failure, hyperlipidemia, tobacco use disorder, and alcohol abuse/alcohol dependency syndrome than controls. 

**Table 1:** 1Demographic characteristics of subjects with stroke and comparison subjects in Taiwan(n=204,372).

Variable	Subjects with stroke (*n*=51,093)		Comparison subjects (*n*=153,279)		*p* value
	Total no.	Percent (%)	Total no.	Percent (%)	
Age (years)					>0.999
18~39	3,220	6.3	9,660	6.3	
40~44	1,893	3.7	5,679	3.7	
45~49	3,078	6.0	9,234	6.0	
50~54	4,390	8.6	13,170	8.6	
55~59	5,118	10.0	15,354	10.0	
60-64	5,238	10.3	15,714	10.3	
65-69	6,147	12.0	18,441	12.0	
≥70	22,009	43.1	66,027	43.1	
Sex					>0.999
Male	27,146	53.1	81,438	53.1	
Female	23,947	46.9	71,841	46.9	
Monthly income					<0.001
NT$1~15,840	25,870	50.6	71,676	46.8	
NT$15,841~25,000	18,547	36.3	56,466	36.8	
≥NT$25,001	6,677	13.1	25,137	16.4	
Geographic region					<0.001
Northern	22,712	44.5	68,304	44.6	
Central	12,630	24.7	36,866	24.1	
Southern	14,032	27.4	42,880	27.9	
Eastern	1,719	3.4	5,229	3.4	
Hypertension	30,494	59.7	66,408	43.3	<0.001
Diabetes	14,840	29.1	30,228	19.7	<0.001
Hyperlipidemia	16,017	31.4	37,984	24.8	<0.001
Tobacco use disorder	2,014	3.9	5,440	3.5	<0.001
Alcohol abuse/alcohol dependence syndrome	302	0.6	465	0.3	<0.001
Coronary heart disease	15,465	30.3	32,013	20.9	<0.001
Atrial fibrillation	1,226	2.4	2,436	1.6	<0.001
Heart failure	2,440	4.8	3,691	2.4	<0.001

Table 2 presents the prevalence of prior IDA between cases and controls. A total of 3,685 study subjects (1.80%) had been diagnosed with IDA prior to the index date; 1,268 (2.48%) were cases and 2,417 (1.58%) were controls. The ^χ2^ test revealed that there was a significant difference in the prevalence of prior IDA between cases and controls (*p* < 0.001).In addition, we found that in the study sample, 2.56% and 1.15% of females and males had IDA respectively. In addition, 1.45%, 2.34%, 2.35%, 1.79%, 1.13%, 1.12%, 1.37%, and 2.26% of the aged groups 18~39, 40~44, 45~49, 50~54, 55~59, 60~64, 65~69, and ≥70 had IDA respectively.

**Table 2:** Prevalence rate and odds ratios(ORs) for iron-deficiencyanemia among sampled subjects.

Presence of prior iron-deficiency anemia	Total (*n*=204,372)		Subjects with stroke (*n*=51,093)		Comparison subjects(*n*=153,279)	
	No.	Percent (%)	No.	Percent (%)	No.	Percent (%)
Yes	3685	1.80	1268	2.48	2417	1.58
Crude **^*a*^**OR (95% CI)	–		1.58*** (1.47~1.69)		1.00	
Adjusted **^*b*^**OR (95% CI)	–		1.49*** (1.39~1.60)		1.00	

*** *p*<0.001.CI, confidence interval, **^*a*^**ORs were calculated by using conditional logistic regression (stratified on sex and age). ***^b^*** Adjusted for patient’s monthly income, geographic region, hypertension, diabetes, hyperlipidemia, tobacco use disorder, alcohol abuse/alcohol dependence syndrome, coronary heart disease, heart failure, and atrial fibrillation.

Furthermore, conditional logistic regression (conditioned on sex, age group, and index year) shows that the OR of prior IDA for cases was 1.58 (95% CI: 1.47~1.69, *p* <0.001) compared to controls. After adjusting for monthly income, geographic region, hypertension, diabetes, CHD, atrial fibrillation, heart failure, hyperlipidemia, tobacco use disorder, and alcohol abuse/alcohol dependency syndrome, the OR of having previously received an IDA diagnosis among cases was 1.49 (95% CI: 1.39~1.60; *p* < 0.01) that of controls.

Table 3 further presents the OR for prior IDA by stroke type. The adjusted OR of prior IDA for cases with ischemic stroke was found to be 1.45 (95% CI: 1.34~1.58) compared to controls. However, we did not find any significant relationship between IDA and subarachnoid/intracerebral hemorrhage even adjusting for other confounding factors (OR=1.17, 95% CI=0.97~1.40). 

**Table 3:** Odds ratios (ORs) of prior iron-deficiency anemia by stroke subtype among sampled patients.

Presence of prior iron-deficiency anemia	Subjects with stroke		Comparison subjects	
	No.	Percent (%)	No.	Percent (%)
Subarachnoid/Intracerebral hemorrhage	*n*=6,614		*n*=153,279	
Yes	120	1.81	2,417	1.58
Crude **^*a*^**OR (95% CI)	1.15 (0.95~1.38)		1.00	
Adjusted **^*b*^**OR (95% CI)	1.17 (0.97-1.40)		1.00	
Ischemic stroke	*n*=31,794		*n*=153,279	
Yes	782	2.46	2,417	1.58
Crude **^*a*^**OR (95% CI)	1. 57*** (1.44~1.70)		1.00	
Adjusted **^*b*^**OR (95% CI)	1.45*** (1.34~1.58)		1.00	
Unspecified strokes	*n*=12,685		*n*=153,279	
Yes	366	2.89	2,417	1.58
Crude **^*a*^**OR (95% CI)	1.84*** (1.65~2.06)		1.00	
Adjusted **^*b*^**OR (95% CI)	1.74*** (1.56~1.95)		1.00	

*** *p*<0.001; CI, confidence interval; ***^a^***ORs were calculated by using conditional logistic regression (stratified on sex and age); ***^b^***Adjusted for patient’s monthly income, geographic region, hypertension, diabetes, hyperlipidemia, tobacco use disorder, alcohol abuse/alcohol dependence syndrome, coronary heart disease, heart failure, and atrial fibrillation.

We also computed the OR for prior IDA by ischemic stroke type. Table 4 shows that the adjusted OR of prior IDA cases with cerebral thrombosis and cerebral embolism were 1.79 (95% CI=1.44-2.24) and 1.90 (95% CI=1.31-2.76), respectively, compared to controls.

**Table 4:** Odds ratios (ORs) of prior iron-deficiency anemia by ischemic stroke subtype among sampled patients.

Presence of prior iron-deficiency anemia	Subjects with stroke		Comparison subjects	
	No.	Percent (%)	No.	Percent (%)
Cerebral thrombosis	*n*=2,695		*n*=153,279	
Yes	84	3.12	2,417	1.58
Crude **^*a*^**OR (95% CI)	2.00*** (1.60~2.49)		1.00	
Adjusted **^*b*^**OR (95% CI)	1.79*** (1.44-2.24)		1.00	
Cerebral embolism	*n*=895		*n*=153,279	
Yes	29	3.24	2,417	1.58
Crude **^*a*^**OR (95% CI)	2.08*** (1.43~3.01)		1.00	
Adjusted **^*b*^**OR (95% CI)	1.90*** (1.31~2.76)		1.00	

*** *p*<0.001; CI, confidence interval; ***^a^***ORs were calculated by using conditional logistic regression (stratified on sex and age); ***^b^***Adjusted for patient’s monthly income, geographic region, hypertension, diabetes, hyperlipidemia, tobacco use disorder, alcohol abuse/alcohol dependence syndrome, coronary heart disease, heart failure, and atrial fibrillation.

## Discussion

In our study we have revealed the possible relations between the IDA and the ischemic stroke through a nationwide database survey. These findings suggest the possibility of an underlying IDA should be considered in patients with ischemic stroke and also support the need for more aggressive managements of IDA. 

IDA is a common form of anemia that accounts for approximately half of all anemia cases worldwide. It has a prevalence of 2–5% among adult men and post-menopausal women in the developed world [2,4]. The disease is caused by insufficient dietary intake and absorption of iron, and/or iron loss from bleeding which can originate from a range of sources such as the intestinal, uterine or urinary tract. Due to the fact that most patients with anemia are asymptomatic, the true incidence of the IDA might be potentially higher than previously reported [2,5].

Anemia was known to be an important risk factor in the development of many cardiovascular diseases [5-8]. Moreover, if anemia is accompanied with chronic diseases, there are also evidences that related morbidity and mortality would increase [6,26,2,7]. Despite the reasonable speculation that a decrease in hemoglobin might possibly compromise the oxygen-carrying ability of the blood flow and subsequently increase the risk of cerebrovascular or cardiovascular diseases, the relationship between the IDA and the stroke was seldom studied. In 1983, Alexander et al. first reported that a patient developing a right hemiparesis and aphasia, was found to be underlying with IDA and marked thrombocytosis [9]. A few years later, another brain infarction case were reported and thought to have resulted from the thrombocytosis secondary to the IDA [2,8].Despite these peculiar cases, the relative importance of IDA seems to have been overlooked as most researchers have chosen to focus on sickle cell anemia. Even after the publication of Framingham study which seemingly implicated hematocrit as an important risk factor for some cardiovascular diseases, the possible relationships between the IDA and the stroke were still yet to be investigated though a large scaled study [17]. Interestingly several reports have revealed that in pediatric populations the IDA seems to contribute to the development of the stroke [29-32]. Maguire et al. conducted the first case-control study to investigate whether IDA is associated with stroke in young children [3,3].The authors found that children with IDA accounted for more than half of all stroke cases in children without an underlying medical illness, which suggests that IDA is a significant risk factor for stroke in otherwise healthy young children. Later on another study investigating patients ≥ 65 years of age admitted to hospital with transient ischemic attack or first ischemic stroke had revealed that IDA prevalence was significantly higher than published National Health and Nutrition Examination Survey III (NHANES III) estimates for gender-specific age groups ≥ 70 years [3,4]. The authors therefore concluded and suggested that a study investigating IDA as a risk factor for ischemic stroke in elderly patients should be conducted. In concordance with these studies and their suggestion that IDA might have something to do with the ischemic stroke in younger and elderly people, our study’s findings further expands on this literature by suggesting that the influences of IDA might affect a larger population than previously believed.

The possible reasons for the IDA as a risk factor for ischemic stroke could be explained that a decrease of the hemoglobin level in the blood stream would likely result in the compromise of tissue oxygen delivery. Several reports have already indirectly suggested IDA as a risk factor for ischemic stroke [29,34,3,5]. Mount et al. report a series of four young children with ischemic stroke underlying with significant IDA [3,6]. Furthermore, the adverse cerebrovascular and cardiovascular effects as a result of the decrease in hemoglobin concentrations were already well documented [37-33,9]. However a majority of these studies have suggested that the human body is able to tolerate a certain anemic state [3,9].It is suggest that instead of baseline hemoglobin level, the maximum decrease in hemoglobin concentration during the operation was associated with increased risk for post-operative complications [40].

Although the main focus of our study was not on patients suffering from acute blood loss, the results of previously mentioned studies emphasized the importance of the basic role of the blood stream: A fluid bearing hemoglobin that carries oxygen needed by the living tissue. We therefore believe that it is reasonable that this association between IDA and stroke can be explained with the simple hemodynamic and oxygen-delivery hypothesis. The hypothesis is further supported by our findings that in patients with hemorrhagic stroke, the chances of receiving a previously IDA diagnosis is not increased. Our findings were consistent with the field’s understanding that thrombocytopenia and subsequent bleeding tendency, instead of commonly seen thrombocytosis, is rarely encountered in patients with iron deficiency [41].

Another possible mechanism that may explain the association between IDA and stroke is through the secondary thrombocytosis as a result of iron deficiency [42].This mechanism is further supported by our findings that both thrombotic and embolic ischemic stroke were associated with the IDA. It is known that thrombocytosis frequently accompanies the less severe anemia of iron deficiency and may be related to platelet stimulation in a manner analogous to the erythropoietin increase seen in many of the anemic states [43,4,4]. Some researchers have stated that iron deficiency status is considered a risk factor for thrombocytosis and should, wherever possible, be avoided [4,5]. Although the influence of secondary thrombocytosis on ischemic stroke has yet to be determined, the evidence from several case reports have implied the possibility that secondary thrombocytosis could be responsible for the underlying mechanism connecting IDA with ischemic stroke [31,46].

The major limitation of this study, like many of the health insurance database analysis researches, comes with the possibility of the surveillance bias. As mentioned before, the ischemic stroke was found to be comorbid with many disorders like cardiovascular and cerebrovascular events. Even though we have limited the date of the IDA diagnosis to be prior to the diagnosis of ischemic stroke making the diagnosis of the IDA irrelevant to various examinations following the stroke event, it is still possible that the ischemic stroke populations in our study have received significantly more laboratory tests than the control group as a result of the presence of other diseases including hypertension, diabetes, and hyperlipidemia. As shown in our results, while our odds ratio did in fact decline slightly, it still remained statistically significant even after adjusting for factors including hypertension, diabetes, and hyperlipidemia. However even though these adjustments were made, the severity of these co-morbidities may differ, contributing certain errors to our reported results. 

Another possible concern is the lack of treatment information for the IDA patients. It is possible that some of the IDA patients were well treated after the diagnosis and were no longer affect by the iron deficiency status. However this problem tends to reduce the significant differences between the IDA and the control group and decrease the OR of prior IDA for cases with ischemic stroke. Therefore we believe this bias would not affect our conclusions based on the statistically significant differences found. 

This study demonstrated an association between IDA and ischemic stroke. Although the casual relationship between IDA and ischemic stroke remains to be further investigated, we recommend that patients found to have IDA should be more aggressively surveyed and managed for the possible underlying bleeding source and/or iron deficiency status in order to reduce the risk of subsequent ischemic stroke.
