# Correction: Association between Ischemic Stroke and Iron-Deficiency Anemia: A Population-Based Study

**DOI:** 10.1371/journal.pone.0170872

**Published:** 2017-01-20

**Authors:** Yen-Liang Chang, Shih-Han Hung, Wells Ling, Herng-Ching Lin, Hsien-Chang Li, Shiu-Dong Chung

The affiliation for the second author is incorrect. Shih-Han Hung is affiliated with: Department of Otolaryngology, Taipei Medical University Hospital, Taipei, Taiwan; Graduate Institute of Medical Sciences, College of Medicine, Taipei Medical University, Taipei, Taiwan; and Department of Otolaryngology, School of Medicine, College of Medicine, Taipei Medical University, Taipei, Taiwan.
